# Determinants of Common Mental Disorders (CMD) among adolescent girls aged 15-19 years in Indonesia: Analysis of the 2018 National Basic Health Survey Data

**DOI:** 10.1371/journal.pgph.0000232

**Published:** 2022-03-15

**Authors:** Muhammad Asrullah, Monique L’Hoir, Maria-João Paulo, Edith J. M. Feskens, Alida Melse-Boonstra

**Affiliations:** 1 Division of Human Nutrition and Health, Wageningen University and Research, Wageningen, The Netherlands; 2 Biometris, Wageningen University and Research, Wageningen, The Netherlands; University College Dublin, IRELAND

## Abstract

Common Mental Disorders (CMD) are distress conditions which manifest themselves with anxiety, somatic, and depressive symptoms. CMD are highly prevalent in Indonesia especially among adolescents. Adolescent girls have a higher risk to develop CMD than boys. This may be related to anaemia, potentially aggravated by early onset of menstruation. This study aimed to determine the association between haemoglobin concentration and other determinants of CMD among adolescent girls in Indonesia. Data of 1,052 adolescent girls aged 15–19 years old from the Indonesian Basic Health Survey 2018 were analysed. CMD was measured using the Self Reporting Questionnaire (SRQ-20). Principal Component Analysis of main determinants was applied and resulting principal components were investigated as risk factors for CMD. The prevalence of CMD among the study population was 16.5%. Anaemia and Age at Menarche (AAM) were not associated with CMD. Three principal components were significantly associated with higher CMD score: 1) higher parental education, better employment of the father, and living in an urban area (β: 0.16, 95%-CI: 0.02; 0.30); 2) higher consumption of salty foods, high-fat foods, and soft drinks (β: 0.23, 95%-CI: 0.05; 0.40); and 3) having asthma, smoking, and a higher haemoglobin concentration (β: 1.74, 95%-CI: 1.59; 1.89). The strongest clustered associates of CMD among adolescent girls in Indonesia were asthma, smoking status, and higher haemoglobin concentration, whereas anaemia and AAM were not associated. Causality of smoking and diet to CMD could not be disentangled in this cross-sectional study. Our findings imply that adolescent girls who have asthma and smoke, as well as those having parents with higher education and secured occupation, are more likely to have mental disorders.

## Introduction

Common mental disorders comprise a number of distressing conditions characterized by anxiety and depression that are often seen in primary care and community settings. These disorders are highly prevalent in the global population with around 4.4% being depressive and 3.6% having an anxiety disorder [1]. Adolescents are specifically susceptible for mental disorders, and the prevalence of CMD among this age group is increasing rapidly in developing countries to an estimated 10–20% [2]. In Indonesia, the prevalence of depression and other mental health disorders were found to be 6.2 and 10%, respectively, among 15 to 24 year olds in 2018, which is higher than the national average [3].

Patients with psychiatric problems suffer more often from anaemia than the overall population [4]. Anaemia is also consistently reported to be related to depression and other mental disorders in adults [5–7]. The most common cause of anaemia is iron deficiency, but shortage of B-vitamins such as vitamin B12 can also play a role. Loss of oxygen-carrying haemoglobin affects normal brain functioning [8]. In addition, both iron and vitamin B12 are known to be involved in physiological processes affecting mental disorders [9]. Anaemia is common among adolescent girls in view of their higher iron needs to compensate for menstrual loss. Globally, more than 50% of adolescent girls at the age of 12–15 years are reported to be anaemic by WHO [10]. However, besides a few reports, there is a dearth of literature on the association between anaemia and CMD in adolescent girls [11].

The aetiology of CMD is multi-factorial and still not fully understood. Puberty and adolescence are characterized by cognitive, physical, social, and emotional changes, and as such it is a vulnerable period in life during which multiple risk factors for CMD coincide. Mental health disorders in adolescents are shaped to a large extent by social determinants, including socioeconomic status and environmental factors [12]. This also concerns quality of parenting and family characteristics that affect children’s physical and emotional growth [13]. From a nutritional viewpoint, puberty markedly increases energy and protein requirements [14]. However, girls tend to diet, which may cause mood disturbances because of caloric deprivation [15]. Dieting may also increase depressive symptoms because of failure to control body weight [16]. The global prevalence of CMD is higher in girls than in boys, approximately 38% versus 23%, respectively [17]. This is apparently related to menarche, a major milestone in puberty which can be perceived by girls as a startling realization of their own physical development [18]. Circulation of sex steroids and their physical manifestation during menarche, as well as neurological changes during adolescent growth may therefore increase the risk of developing a depression [19]. Early pubertal onset is one of the factors associated with increased risk of CMD around the world [18, 20]. This may be caused by new situations and expectations that girls have to face, leading to shame, distress, and to self-imposed behaviour to keep menstrual status hidden. Experiencing physical changes earlier than peers, such as increases in body mass index, is correlated with detrimental psychological outcomes such as depression and sleep disturbances [21]. It can lead to feelings of sickness and moodiness, hampered school and cognitive performance, psychosomatic symptoms, eating disorders, and lower body satisfaction because of social stigma and peer pressure [22]. Girls that mature early were found to worry more about their menarche, which was correlated with a lower self-esteem, a more external locus of control, and increased anxiety [23].

In this paper, we aim to explore the epidemiologic evidence on anaemia and other potential aetiologic factors as determinants of CMD in Indonesian adolescent girls aged 15–19 years. For this, we analysed data from the Indonesian Basic National Health Survey conducted in 2018. Better understanding of the aetiology of CMD will help us to design preventive interventions and effective programs for adolescent girls in Indonesia.

## Methods

### Ethics statement

All procedures were in accordance with the ethical standards to protect the respondent’s information and privacy. Ethical approval has been issued by the Health Research Ethics Commission, Ministry of Health, Republic of Indonesia No. LB.02.01/2/KE.267/2017, and informed consent was obtained from all participants and their parent. The dataset was obtained from the National Institute of Health Research and Development (NIRD), Ministry of Health, Republic of Indonesia without respondent’s personal information due to data privacy policy, after approval of the proposed data analysis.

### Study design and data collection

The Indonesian Basic Health Survey (*Riskesdas*) is a National Health Survey which represents the Nation, Provinces, and Districts and is conducted every five years on household and individual level. For the present study, the data from *Riskesdas* conducted in 2018 was analysed because of the availability and completeness of CMD data. The *Riskesdas* was established by the National Institute of Health Research and Development, Ministry of Health, Republic of Indonesia. Data collection instruments in 2018 had mostly already been used in previous Basic National Health Surveys (Riskesdas surveys), conducted in 2007, 2010, and 2013. The validation of these instruments was carried out by a variety of researchers, the Research and Development Agency, academics, and professional organizations. During data collection, field supervision was done by provincial technical and managerial teams in order to monitor interviews, to check the data entry and cleaning process, and to check availability of tools [3].

Cascade training was applied from national level to data collectors on district level to have the same perception on content and procedures. Trained staff collected the data using a one-on-one interview through structured questionnaires on household and individual level, and anthropometry measurements. The households were selected randomly through 4 stages of sampling, based on the representativeness of the population and rural-urban distribution [3, 24]. Adolescent girls were recruited from these households with all adolescents in the house being interviewed. Data was collected from 282,654 households, including 1,017,290 household members (the overall recruitment rate of household interviews was 95.58%, while this was 93.2% for individual interviews). For blood collection, a national representative subsample of 24,980 households was selected using a probability proportional to size approach with linear systematic sampling (final sample: 19,418; completion rate: 77.7%). Blood samples were collected by a different team of data collectors who were experienced in medical services. Interviews and blood draws were done for all household members, but only after signing for informed consent. The respondents for the present analysis were female adolescents aged 15–19 years who were interviewed, amongst others, about their age at menarche (AAM), depression and mental health, and of whom haemoglobin concentration was measured (n = 1,274). After removing cases with incomplete data on CMD, AAM, and other individual and parental characteristics, data of 1,052 adolescent girls remained for analysis (S1 Fig).

### Variables

CMD was measured using a self-reporting questionnaire (SRQ-20), a psychometric tool developed by the World Health Organization (WHO) that has been used widely in Low and Middle Income Countries (LMIC) [25]. The SRQ-20 has been adopted by the Indonesian Ministry of Health since the first Riskesdas in 2007, and has been locally validated for use among people 15 years and older with a Positive Predictive Value (PPV) of 60% and a Negative Predictive Value (NPV) of 92% [26]. The Indonesian Ministry of Health with WHO and other researchers have used the data to monitor CMD among all age groups at the national level over time [27–29]. In this questionnaire, CMD is determined by 20 yes/no questions related to mental disorders, divided over four subcategories, namely ‘depression/anxiety’, ‘somatic symptoms’, ‘reduced vital energy’, and ‘depressive thoughts’. We dichotomized the presence or absence of CMD (cut off point ≥ 6) in order to calculate the prevalence of occurrence, and used the continuous CMD score of 0–20 in further analysis to determine associations with potential determinant variables. To see whether the SRQ-20 was reliable in the present study, the reliability coefficient (Cronbach’s alpha) was calculated.

Determinants of CMD considered in this study consisted of both adolescent-related and environmental factors (S1 Table). Adolescent-related factors were anaemia status, age at menarche (AAM), Body Mass Index (BMI)-for-age z-score, adolescent occupation, iron supplementation, smoking status, diagnosed respiratory tract infection and asthma, and consumption of unhealthy foods. Environmental factors were factors related to household and living circumstances such as parental education, parental occupation, number of household members, and demographic area.

#### Adolescent factors

Anaemia status was identified based on haemoglobin concentration which was measured by finger prick method using a portable HemoCue 201+ machine. A haemoglobin concentration <12.0 g/dl was classified as anaemia. AAM was determined by two questions in a structured questionnaire, namely “Have you started to menstruate?” followed by a question “How old (years) were you when you had your first menstruation?” AAM was classified into the following age categories: ≤11 years,12–14 years, 15–17 years, and ≥18 years.

Height was measured by microtoise (SECA 206) and weight by a flat digital weighing scale. BMI-for-age z-score was classified into five categories namely severe thinness (< -3 SD), thinness (≥ -3 SD ≤ -2 SD), normal (>-2 SD to ≤1 SD), overweight (>1 SD to ≤2 SD), and obesity (>2 SD). Smoking status was labelled into “yes” if a respondent smoked every day or sometimes, while respondents who had no smoking experience were labelled into “no” (i.e. non-smoker). Information on any diagnosed communicable and non-communicable diseases was included in the standardized questionnaire by asking the respondent:” Have you been diagnosed with (disease) by medical doctors?” For most diseases the prevalence was 0% among the adolescent girls. Therefore, only two diseases were retained in the analysis: respiratory tract infections and asthma. Unhealthy food consumption, which refers to consumption of salty food, high-fat food, and soft drinks, was determined by a short questionnaire. Each food category was classified based on frequency of consumption with “high” for ≥1 time per day, “moderate” for 1–6 times per week, and “low” for ≤ 3 times per month.

#### Household factors

Parental education was classified for both parents into 4 categories namely “no education”, “elementary school”, “senior high school”, and “diploma program or higher” if they finished the indicated level of education. With respect to the “no education” group, fathers and/or mothers who were never enrolled in education, or enrolled but did not finish elementary school, were categorized into this category. Parental occupation was classified for both parents into 3 groups, namely unemployed, unsecured job, and secured/paid job. Fathers and mothers who had no job/work were classified as being unemployed, while those who worked as temporary employee in a private company, as entrepreneur, farmer, fisherman, worker, craftsman, driver, or as a housekeeper were classified as having an unsecured job. Furthermore, fathers and mothers were classified as having a secured job if they worked as civil servant, army or police officer, worked for state companies, or worked as a driver or housekeeper with a monthly based salary. The number of household members was classified into 2 categories, namely adolescents who lived in a home together with < 5 people or with ≥ 5 people. Demographic area was divided into rural and urban using the classification of Indonesia’s Bureau of Statistics, which is based on population density, percentage of agricultural households, and presence/access to facilities.

### Statistical analysis

All data analysis was conducted with STATA statistical software, version 16 (StataCorp LCC, TX, USA). Complete case analysis was employed, with minor differences between excluded and included cases; excluded cases tended to be slightly more often member of a smaller household, living in urban areas, have a highly educated father and a mother without education (see S2 Table for missing data analysis). Prevalence and estimation of the proportion of the population having CMD was analysed by using the Survey (*Svy*) command in STATA to adjust for sampling weights. In Riskesdas, sampling weights at National, Provincial, and District level were generated by adjusting for sampling procedures as well as for intra-household dependency. Both discrete and continuous variables, such as haemoglobin concentration, AAM, BMI-for-age z-score, and number of household members, were included in Principal Component Analysis (PCA). We also used categorical variables that were converted into dummy variables. Barlett’s test of sphericity was used for the correlation matrix between determinant variables and CMD score. PCA was conducted with, firstly, a scree-test to assess the number of components, which were each explained by eigen values. Secondly, an orthogonal component rotation (varimax) was examined to more clearly distinguish all determinant variables that correlated highly with each component and to aid interpretation. Principal Component Regression (PCR) of CMD as continuous variable (i.e. linear regression) was applied on the resulting principal components. Multiple regression of CMD on the original variables that were part of the components with a high variance proportion in PCR, was employed to see their interactions and understand how these variables were associated with CMD.

## Results

The scale reliability coefficient (Cronbach’s alpha; all indicators) of the SRQ-20 questionnaire was 0.866, indicating good internal consistency. Data from the total of 1,052 included adolescent girls aged 15–19 years show that the anaemia prevalence was 27.3%. With respect to food consumption, 49.9% and 41.0% adolescents consumed high-fat food and salty foods ≥1 time per day, respectively. Approximately half of the fathers and 45% of mothers had at least completed secondary school, but the majority of mothers were unemployed.

The mean total CMD score was 4.0 (SD: 2.4). Based on CMD classification, the prevalence of CMD was 16.5% (Table 1). Among the 287 adolescents with anaemia, 16.8% were suspected to have CMD and this did not differ from those without anaemia (16.4%, p = 0.87). Although the prevalence of CMD showed a downward trend with age at menarche, differences between categories were not statistically significant (p = 0.59). Prevalence of CMD was significantly higher in adolescents with overweight (22.9%), who smoked (42.2%), who had asthma (40.1%), and who consumed more often salty foods or soft drinks (19.9%). Furthermore, the prevalence of CMD was lower in the group who had a father with only elementary school education (12.6%), and higher in adolescents living in an urban area (19.1%). Among the four subcategories of the SRQ-20 questionnaire, girls with CMD indicated most often to ‘feel nervous, tense or worried’ (17.8%) in the subcategory ‘depression/anxiety’, to ‘often have headaches’ (32.6%) within the category of ‘somatic symptoms’, to be ‘easily tired’ (19.3%) in the category of ‘reduced vital energy’, and to have ‘lost interest in things’ (6.1%) in the category of ‘depressive thoughts’ (S3 Table).

**Table 1:** Prevalence of CMD^1^ among Indonesian girls aged 15–19 years old according to adolescent-related and household factors (Riskesdas 2018).

Variables		Prevalence^2^		
Adolescent factors	N	n	% (95% CI)	p-value^3^	
**All**	1,052	169	16.5 (14.3; 18.8)	0.000	
**Anaemia status**					
No	765	122	16.4 (13.4; 18.7)	0.876	
Yes	287	47	16.8 (12.3; 21.2)		
**Age at Menarche (years)**					
9–11	86	16	21.5 (11.1; 28.5)	0.595	
12–14	834	136	16.2 (13.9; 18.9)		
15–17	130	17	15.4 (7.7; 19.8)		
≥ 18	2	0			
**Nutritional status (BMI z-score)**					
Severe thinness	280	41	15.5 (10.7; 19.4)		
Thinness	134	15	11.2 (6.4; 17.8)	0.158	
Normal	361	68	18.4 (14.9; 23.3)		
Overweight	65	14	22.9 (12.3; 33.4)		
Obese	212	31	16.5 (10.2; 20.2)		
**Adolescent Occupation**					
Unemployed	277	39	14.4 (10.2; 18.7)	0.606	
Student	679	116	17.4 (14.3; 20.2)		
Work (Paid and unpaid)	96	14	16.8 (8.2; 23.3)		
**Iron Supplementation**					
Not supplemented	906	146	17.0 (13.8; 18.7)	0.286	
Supplemented (<52 pills)	146	23	13.3 (10.3; 22.7)		
**Smoking Status**					
Not Smoking	1,028	161	15.9 (13.5; 18.1)	0.002	
Smoking	24	8	42.2 (15.6; 55.4)		
**Diseases diagnosed**					
Respiratory infection					
No	1,019	6	16.3 (13.8; 18.4)	0.400	
Yes	33	163	22.6 (6.9; 35.5)		
Asthma					
No	1,021	159	15.7 (13.4; 17.9)	0.002	
Yes	31	10	40.1 (16.7; 51.4)		
**Risk consumption**					
Salty food					
High	431	31	19.9 (9.6; 19.1)	0.027	
Moderate	397	57	14.6 (11.1; 18.1)		
Low	224	81	11.9 (15.2; 22.8)		
High-fat food					
High	525	76	15.4 (11.6; 17.8)	0.356	
Moderate	416	77	18.7 (14.9; 22.6)		
Low	111	16	13.8 (8.5; 22.4)		
Soft drink					
High	28	8	36.0 (13.2; 48.7)	0.008	
Moderate	171	34	21.2 (14.2; 26.7)		
Low	853	127	14.9 (12.6; 17.5)		
**Household factors**					
**Paternal education**					
No education	40	6	20.9 (5.7; 29.8)	0.047	
Elementary school	473	61	12.6 (10.1; 16.3)		
Senior high school	469	90	19.8 (15.7; 23.1)		
University	70	12	19.7 (9.2; 28.1)		
**Maternal education**					
No education	39	6	20.5 (5.9; 30.5)	0.301	
Elementary school	532	78	14.4 (11.8; 18.0)		
Senior high school	419	77	19.2 (14.8; 22.4)		
University	62	8	16.5 (5.7; 23.9)		
**Paternal occupation**					
Unemployed	20	3	15.5 (3.2; 37.9)	0.834	
Unsecured job	589	92	15.8 (12.8; 18.8)		
Secured Job	443	74	17.3 (13.4; 20.5)		
**Maternal occupation**					
Unemployed	522	78	15.8 (12.0; 18.3)	0.317	
Unsecured job	389	63	15.8 (12.7; 20.3)		
Secured job	141	28	21.3 (13.6; 27.4)		
**Number of household members**					
<5	487	70	14.9 (11.4; 17.8)	0.231	
≥ 5	565	99	18.0 (14.5; 20.9)		
**Demographic area**					
Rural	444	56	12.2 (9.7; 16.0)		
Urban	608	113	19.1 (15.6; 21.9)	0.006	

^**1**^Cut-off for having suspected CMD was a score of ≥6 points out of 20.

^2^All prevalence and population proportions of CMD were estimated using weighted data.

^3^Differences between proportions of adolescent with suspected CMD were tested by chi-square/ Fisher exact.

Based on eigenvalues, difference, proportion, and cumulative variance, we decided to extract 6 components for the entire sample which explained 45.5% of the CMD variance in the sample (S4 Table). It was considered reasonable to retain all components with eigenvalues greater than 1. Rotation (orthogonal) was used to maximize the variance captured by the first component, so that the selected components could explain maximum variance in the data set (S2 Fig). Each symptom of CMD was given a score for each component. In Table 2, we show the most important factor loadings of each of the six components. For clarity, we do not display small loadings (absolute value smaller than 3). The first component was characterized most strongly by parental education, and, although to a lesser extent, also by paternal occupation and living area (Table 2). It represents socio-economic and demographic determinants. The second component was representative of unhealthy food consumption which was characterized most prominently by salty food and high-fat food, and less so by soft drink consumption. The third component represented health and lifestyle factors, including haemoglobin concentration, smoking status, and asthma. The factor asthma was most prominent among the three.

**Table 2:** Results of PCA of selected characteristics among Indonesian girls aged 15–19 years old (Riskesdas 2018).

Items	Comp. 1	Comp. 2	Comp. 3	Comp. 4	Comp. 5	Comp. 6
Haemoglobin			0.325		0.370	
Age At Menarche				-0.586		
BMI z-score				0.574		
Occupation						
Iron Supplementation						0.658
Smoking status			0.410		-0.397	
Respiratory infection				-0.396		
Asthma			0.516			
Salty food consumption		0.605				
High fat food consumption		0.637				
Soft drink consumption		0.304				-0.446
Paternal education	0.585					
Maternal education	0.581					
Paternal occupation	0.349					
Maternal occupation					0.458	0.312
Number of household members					-0.561	
Living area (rural-urban)	0.412					
Variance proportion (%)	11.70	7.37	6.98	6.80	6.47	6.11
Cumulative (%)	45.44					

The last three components were composed of AAM (negative factor loading), BMI-for-age z-score, and respiratory tract infection (negative) (Component 4); health, socio-economic status, haemoglobin concentration, smoking status (negative), parental occupation, and number of household members (negative) (Component 5); and iron supplementation, soft drink consumption (negative), and maternal occupation (Component 6).

Principal Component Regression (PCR) was done using all components with CMD score as an outcome (Table 3). Based on this, only the first three components were significantly associated with CMD. Among these, component 3 had the strongest association with CMD score and explained the highest proportion of the variance in comparison to components 1 and 2. We did not find any significant associations between component 4, 5, or 6 and CMD. Several models were constructed including combinations of the principal components as explanatory factors to select the best model based on its goodness-of-fit. The analysis showed that the model only including component 3 had the best data fit (S5 Table). Additional analysis was employed for a more detailed picture of the interrelation between variables in component 3 (Table 4). Among the variables in Component 3, the interaction between asthma and smoking status was significant and strongly associated with CMD score, suggesting that teenagers who smoke while suffering from asthma have the largest risk for CMD. Haemoglobin concentration was positively associated with CMD in girls who had asthma or who smoked.

**Table 3:** Results of principal component regression of six retained components.

Component	β coefficient	(95% CI)	P-value	R^2^
**Component 1**	0.16	0.02; 0.30	0.022*	0.005
Paternal education				
Maternal education				
Paternal occupation				
Living area				
**Component 2**	0.23	0.05; 0.40	0.016*	0.005
Salty food consumption				
High fat consumption				
Soft drink consumption				
**Component 3**	1.74	1.59; 1.89	0.000*	0.337
Haemoglobin				
Smoking status				
Asthma				
**Component 4**	0.16	-0.02; 0.34	0.091	0.003
Menarche				
BMI z-score				
Respiratory infection				
**Component 5**	0.18	-0.07; 0.37	0.854	<0.001
Haemoglobin				
Smoking status				
Mother occupation				
Number of household members				
**Component 6**	<0.01	-0.20; 0.19	0.975	<0.001
Iron supplementation				
Soft drink consumption				
Maternal occupation				

**Table 4:** Results of linear regression of asthma, smoking and Hb.

Variables	β coefficient		
	Estimated Effect	(95% CI)	P value
Asthma (yes)	1.67	0.47; 2.87	0.006
Smoking (yes)	1.88	0.52; 3.24	0.007
Haemoglobin (g/dL)	0.05	-0.6; 0.18	0.330
Asthma (yes) and haemoglobin^1^	0.18	0.04; 0.32	0.016
Smoker (yes) and haemoglobin^2^	0.22	0.06; 0.37	0.008
Asthma (yes) and Smoker (no)^3^	1.32	0.09; 2.56	0.036
Asthma (no) and Smoker (yes)^4^	1.44	0.03; 2.86	0.046
Asthma (yes) and smoker (yes)^5^	7.17	2.53; 11.81	0.003

Notes:

^1^the estimated effect is the slope of haemoglobin for adolescents with asthma, relative to the slope of haemoglobin estimated for adolescents without asthma

^2^the estimated effect is the slope of haemoglobin for smokers, relative to the slope of haemoglobin estimated for non-smokers

^3^the estimated effect is relative to adolescents with asthma who do not smoke

^4^the estimated effect is relative to adolescents without asthma who do smoke

^5^the estimated effect is relative to adolescents who do not have asthma and who do not smoke.

## Discussion

The current study aimed to determine the association between CMD and determinants of health among adolescent girls aged 15–19 years in Indonesia. The prevalence of mental health disorders was found to be 16.5%, which was higher than the prevalence of 10% reported for all 15 to 24 year olds [3]. This is consistent with the fact that prevalence of CMD among younger age groups in developing countries is growing [2]. Furthermore, the findings of this study imply that CMD occurs more often among adolescents who have parents with a higher socio-economic status and who are living in an urban environment, in addition to those having asthma. This study also suggests that AAM and anaemia were not linked to the occurrence of CMD.

We found that smoking status, asthma, salty food consumption, paternal education, and demographic area were associated with CMD in univariate analysis. This was in line with the PCA showing that having asthma, being a smoker, and having higher haemoglobin concentrations was the primary component to be associated with CMD score. A previous study showed that asthma caused complications including sleep difficulties, persistent coughing, and limitation of activity [30]. Furthermore, adolescents with asthma were twice as likely to exhibit anxiety symptoms in comparison to their healthy peers [31]. Perception of illness such as the feeling that asthma has a detrimental effect on one’s life and emotions, and worry that it is difficult to control the disease is one of an individual’s cognitive components that might be part of the pathway to explain why asthma may be related to anxiety symptoms [32]. The perception might also be linked to lower self-management and functional status, hospitalization, and increased medical cost [33].

Smoking has also been related to mental disorders previously. For instance, a cohort study in the US showed that adults with at least one psychiatric disorder in their life are three times more likely to be current smokers compared to those without such a disorder [34]. Another study showed that an association between depression and smoking was stronger among women as compared to men [35]. Higher smoking rates were also found specifically among adolescent girls living with mental disorders [36]. A question remains regarding the causality of this association since this cannot be disentangled in cross-sectional studies.

In the current study, anaemia was not independently associated with CMD, but haemoglobin concentration correlated positively in conjunction with asthma and smoking in explaining CMD score. The finding of higher haemoglobin among people with asthma is in line with a previous study [37]. Asthma may lead to increased binding of carbon monoxide to haemoglobin or to the formation of methaemoglobin by nitric oxide (NO), resulting in lower oxygen capacity and increasing NO concentration. This prevents oxygen to bind to haemoglobin and therefore leads to hypoxia, which can lead to increased haemoglobin concentration [38]. The longer hypoxia caused by breathing problems remains uncontrolled, the more likely that it will cause stress and anxiety in asthmatic patients. Although haemoglobin levels are not significantly different among adolescent smokers and non-smokers in this study, higher haemoglobin levels have been found in smokers compared to non-smokers because of the same hypoxia hypothesis [39].

Another component that we found to be related to CMD was consumption of salty food, high-fat food, and soft drinks. Unhealthy eating patterns including consumption of junk food and snacking behaviour have previously been associated with higher prevalence of mental disorders among adolescents [40]. Another study showed that westernized dietary patterns, such as diets characterized by high intake of saturated fat, processed meat and sodium, and low consumption of vegetables, were associated with depression and anxiety [41]. Also, high consumption of sugared beverages and sweet foods has been associated with several mental health problems [42]. A biological explanation for this could be that unhealthy diets low in micronutrients, such as magnesium, zinc, and folate, would be associated with depression and anxiety [43]. Another pathway could be a direct effect of unhealthy food on inflammatory parameters, oxidative stress, and the immune system which has a proven link with mental disorders [44]. A diet high in sugar was also found to be involved in neural pathways which impact low basal levels of dopamine, particularly in the nucleus accumbent. The consumption of sucrose triggers the mesocorticolimbic system in which morphological neural changes, emotional, and behavioural processing are impaired [45]. It might also be the other way around, however, that girls with CMD tend to consume unhealthy foods especially those with higher social economic status. However, a previous prospective study conducted among Australian adolescents did not support this reverse causality hypothesis [46]. Further prospective research would be needed to unravel the direction of the relationship between CMD and unhealthy food habits.

We also found that parents with a high level of education, a father with a secured job, and living in an urban area were components that were significantly associated with a higher CMD score. Although poverty, including unemployment, inferior housing, and low income have consistently been associated with increased prevalence of mental illness among adults, we found quite the opposite among these adolescent girls. This may be explained in various ways. Urbanicity is known as a rather independent risk factor of mental health problems among adolescents [47] with clear evidence of causality [48]. Adolescents who lived in urban areas were reported previously to have biological stress reactivity, expressed as the difference between cortisol concentration and heart rate during rest and during stress [49], and were more likely to have symptoms of depression, especially females [50]. In urban areas, greater psychosocial stress due to crowded environments and lack of social cohesion may also trigger the limbic system in the human brain negatively, expressed by higher neuronal glucocorticoid receptor expression and lower neuropeptide oxytocin. Furthermore, dysregulated systemic stress functioning, such as acute autonomic nervous system and hypothalamic-pituitary-adrenal axis reactivity and functioning measured by adolescents’ heart rate and salivary cortisol level, was found among people who grew up in urban areas compared to rural areas [47].

In addition, higher socioeconomic status and living in an urban area may expose adolescents to less quality time with their parents which may make them more susceptible to mental health problems. Reversely, those with lower socio-economic status or living in rural areas may experience more social cohesion both within households as well as in the community [51]. Maintaining support, contact, everyday activities at home, and forming supportive peer relationships may lower the risk of CMD in adolescents [52]. A previous study conducted in a representative sample of Korean adolescents, showed that those who had a father with a better occupation, and a high work load were more likely to have depression and suicidal thoughts [53]. Moreover, the risk of mental health problems among Japanese children aged 4–12 years old increased when parents worked and returned home late or had irregular working hours [54]. Another study demonstrated that risk of adolescent depression was increased by less closeness and less time togetherness between parents and adolescents aged 13 and 14 years old [55]. The frequency of parent-child contact was found to be a protective factor against negative psychosocial outcomes among adolescents aged 10–15 years old [56].

Despite many findings stating that higher income reduces the risk of mental health problems as a consequence of financial stress and social well-being [57, 58], our finding is in line with a previous study conducted in Indonesia that showed higher probability of depression among the high-income group of patients with psychiatrist-diagnosed clinical depression [59]. An alternative explanation could be that parents with higher education have higher expectations towards their children’s achievements, and thereby put more pressure on them. However, the available dataset did not allow us to explore this or any of the other explanations described above, since no data were collected on covariates such as family income, family expenses, socio-cultural status and cohesion, adolescent’s experience of violence and quality of their relationship with parents and peers. Follow up studies will therefore be needed to further unravel this association.

This study was done with national representative data from Indonesia and included a comprehensive set of both social and health determinant factors. Self-reporting, as used in this survey, has previously been shown to be an effective method to evaluate certain subjective experiences such as depressive symptoms [60]. This study has some limitations. The analysis lacked covariates that could explain some of the associations found in more detail. Nevertheless, socioeconomic status was captured by parental education and occupation [24]. Furthermore, the cumulative variance of the PCA was low. Missing data analysis revealed a potential bias towards exclusion of cases that were more often member of a smaller household, lived in urban areas, had a highly educated father and a mother without education and tended to have CMD more often, which may have blunted some of the associations. Recall bias from self-reported CMD data might have led to misclassification. Lastly, the cross-sectional nature of the study prohibits causal inferences, and the time effect cannot be determined. Nevertheless, the findings provide valuable information for follow up and policy decision making regarding adolescent mental health in Indonesia.

## Conclusion

The strongest clustered associates of CMD among adolescent girls in Indonesia are asthma, smoking, and haemoglobin concentration, followed by consumption of unhealthy foods and higher socio-economic status. The causality of smoking and diet to CMD cannot be disentangled due to the cross-sectional study design. Anaemia and earlier age at menarche were not associated with CMD. This study implies that adolescent girls with asthma and smoking cigarettes, as well as those having higher parental education and secured occupation, have an increased risk of developing mental disorders.

## Supporting information

S1 Fig Data cleaning process. (PDF)

S2 Fig a) Scree plot of eigenvalues after PCA; b) Biplot of PCA.
(PDF)

S1 Table Variables: Definition, unit, classification, score, survey question and descriptive analysis. (PDF)

S2 Table Prevalence comparison between final dataset, complete data based on variables, and excluded respondents among Indonesian girls aged 15–19 years old. (PDF)

S3 Table Subdivision of CMD symptoms among girls aged 15–19 years old from Riskesdas data 2018. (PDF)

S4 Table Eigen values of principal component in CMD. (PDF)

S5 Table Model fit of principal component regression. (PDF)
