# Supplementary figures and images for: Determinants of Common Mental Disorders (CMD) among adolescent girls aged 15-19 years in Indonesia: Analysis of the 2018 National Basic Health Survey Data
Source: PLOS Glob Public Health. 2022 Mar 15;2(3):e0000232. doi: 10.1371/journal.pgph.0000232 (PMC10021533; doi:10.1371/journal.pgph.0000232)

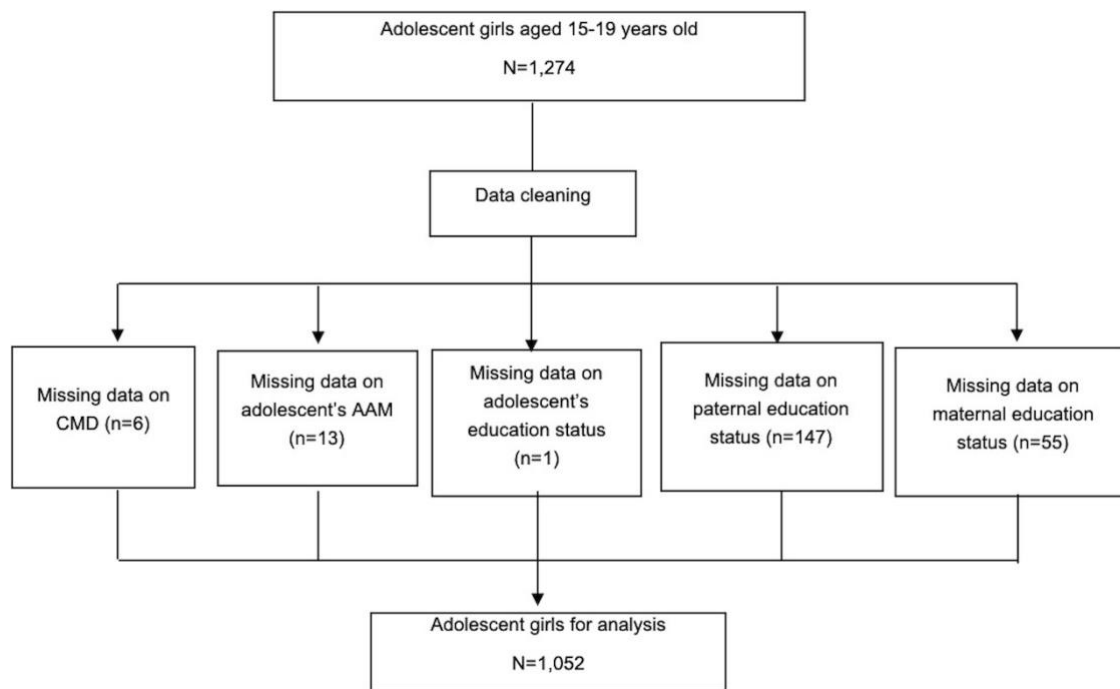

S1 Fig. Data cleaning process

Supplement: S1 Fig — (PDF) [file pgph.0000232.s001.pdf]

a)

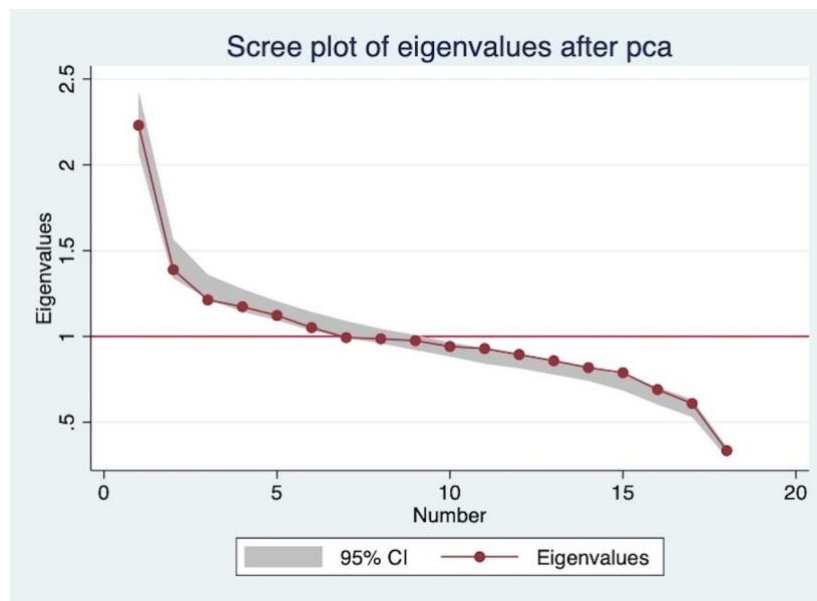

b)

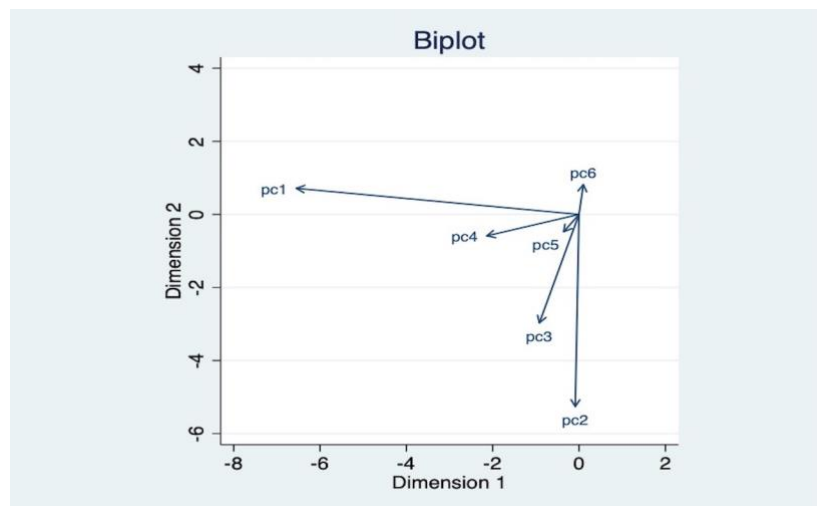

S2 Fig. a) Scree plot of eigenvalues after PCA; b) Biplot of PCA

Supplement: S2 Fig — a) Scree plot of eigenvalues after PCA; b) Biplot of PCA. (PDF) [file pgph.0000232.s002.pdf]
