# Supplementary material for: Determinants of Common Mental Disorders (CMD) among adolescent girls aged 15-19 years in Indonesia: Analysis of the 2018 National Basic Health Survey Data
Source: PLOS Glob Public Health. 2022 Mar 15;2(3):e0000232. doi: 10.1371/journal.pgph.0000232 (PMC10021533; doi:10.1371/journal.pgph.0000232)
Supplement: S1 Table — (PDF) [file pgph.0000232.s003.pdf]

S1 Table. Variables: definition, unit, classification, score, survey question and descriptive analysis

Variable	Description of variables/ definition	Question/ measurement	Unit	Answering options	Classification
**Adolescent variables**					
CMD	CMD refers to anxiety and depression which impacts mood or feelings of affected adolescents	Individual 20-item questionnaire: *'In the past 2 weeks, have you experienced/felt any of the following symptoms?'*	Total score (0-20)	1: No, score = 0<br>2: Yes, score = 1	0: Total score <6: No CMD<br>1: Total score ≥ 6 CMD
Anaemia	Anaemia is a condition in which the number of red blood cells or haemoglobin concentration is lower than normal	Hemocue +201	Haemoglobin (g/dL)	-	0: Haemoglobin ≥12 g/dL: no anaemia<br>1: Haemoglobin <12 g/dL: anaemia
Age at Menarche (AAM)	AAM is the age when menarche or first menstruation occurs	Individual questionnaire: *'How old were you when you had the first menstruation?'*	Years of age	-	1: ≤11 years old<br>2: 12-14 years old<br>3: 15 – 17 years old<br>4: ≥ 18 years old
Nutritional status	Nutritional status is adolescent's weight status indicated by BMI-for age-z-score	Height (cm) and weight (kg)	BMI-for-age Z-score	-	1: ≤ -3SD (Severe thinness)<br>2: >-3SD ≤ -2SD (Thinness)<br>3: >-2SD < 1SD (Normal)<br>4: >1 SD to ≤2 SD (Overweight)<br>5: >2 SD (Obese)
Adolescent's Occupation	Occupation refers to activities in which adolescents engage when pursuing their pleasure on a daily basis	Individual questionnaire: *'What is your occupation?'*		1. No work<br>2. School<br>3. Civil servant, army, police, governmental organization<br>4. Non-governmental organization<br>5. Entrepreneur<br>6. Farmer<br>7. Fisherman<br>8. Driver/ housekeeper<br>9. Other	1: Unemployed (if 1)<br>2: Student (if 2)<br>3: Work (paid or unpaid) (if 3-9)

Iron supplementation	Iron supplementation refers to the number of *iron* supplements that are consumed by adolescents to treat and prevent *iron* deficiency and anaemia, either received from a government program or bought	Individual questionnaire: *'Have you received / bought an iron supplement in the past 12 months?*'		1. Yes<br>2. No	0: Not supplemented (if 2)<br><br>1: Supplemented (if 1)
Smoking Status	Smoking status is a recoded variable based on a question about cigarette smoking	Individual questionnaire: *'Do you smoke?'*		1. Yes, every day<br>2. Yes, sometimes<br>3. No	0: Not smoking (if 3)<br><br>1: Smoking (if 1, 2)
Diagnosed diseases	Diagnosed diseases refer to all diseases that have been diagnosed by a medical doctor	Individual questionnaire: *'Have you been diagnosed with (disease) by a medical doctor?'*		1. Yes<br>2. No	1: Never been diagnosed (if 2)<br><br>2: Have been diagnosed (if 1)
Unhealthy food consumption	Unhealthy food consumption refers to consumption of salty food, high-fat food, and soft drink	Food frequency questionnaire:<br><br>*'In the past month, how many times did you consume (food)?'*	Frequency of consumption	1. > 1 time per day<br>2. 1 time per day<br>3. 3 – 6 times per week<br>4. 1 – 2 times per week<br>5. < 1 time per month<br>6. Never	1: Low (if 4, 5, 6)<br><br>2: Moderate (if 3)<br><br>3: High (if 1, 2)
**Household variables**					
Parental education	Parental education is defined as the highest education level of parents attained	Household questionnaire: *'What is your (father/ mother) last education'*		1. Never attended school<br>2. Elementary school (not completed)<br>3. Elementary school (completed)<br>4. Middle school (completed)<br>5. Senior high school (completed)<br>6. Diploma (completed)<br>7. University (completed)	1: No education (if 1, 2)<br><br>2: Elementary school (if 3,4)<br><br>3: Senior high school (if 5)<br><br>4: Diploma or higher (if 6,7)
Parental occupation	Parental occupation is defined as activities in which parents engage when pursuing their pleasure on a daily basis to have income	Household questionnaire: *'What is your (father/ mother) occupation?'*		1. No work<br>2. School<br>3. Civil servant, army, police, governmental organization<br>4. Non-governmental organization<br>5. Entrepreneur<br>6. Farmer<br>7. Fisher<br>8. Driver/ housekeeper<br>9. Other	1: Unemployed (if 1, 2)<br><br>2: Unsecured job (if 5, 6, 7, 9)<br><br>3: Secured job (if 3, 4, 8)

Number of household members	Number of household members is defined as the number of people living in the same building, not considering the household registration	Household questionnaire*: 'How many people live in this house/ building (not based on household)?'*	Total number of people		1: ≤ 5 people<br>2: > 5 people
Demographic area	Demographic area is defined as the classification of the living area based on the percentage of agricultural households, and the presence/access to facilities, as determined by Indonesia's Bureau of Statistics	Assessed by data collectors based on area criteria		1. Urban area<br>2. Rural area	1: Rural (if 2)<br>2: Urban (if 1)
