# Supplementary material for: Determinants of Common Mental Disorders (CMD) among adolescent girls aged 15-19 years in Indonesia: Analysis of the 2018 National Basic Health Survey Data
Source: PLOS Glob Public Health. 2022 Mar 15;2(3):e0000232. doi: 10.1371/journal.pgph.0000232 (PMC10021533; doi:10.1371/journal.pgph.0000232)
Supplement: S2 Table — (PDF) [file pgph.0000232.s004.pdf]

S2 Table. Prevalence comparison between final dataset, complete data based on variables, and excluded respondents among Indonesian girls aged 15-19 years old

	All cases		Complete cases		P-value[a]	Excluded cases		P-value[b]
	N	Value	N	Value		N	Value	
AAM, mean years ± SD	1,274	13.04 ± 1.29	1,052	13.01 ± 1.27	0.574	222	13.00 ± 1.09	0.664
CMD, %	1,268	16.9 (14.9; 19.1)	1,052	16.9 (14.9; 19.1)	0.599	216	21.8 (15.8; 26.7)	0.100
Anaemia, %	1,274	27.1 (24.7; 29.7)	1,052	27.3 (24.5; 29.9)	0.912	222	26.1 (20.5; 32.5)	0.784
Urban, %	1,274	59.1 (56.4; 61.8)	1,052	57.8 (54.8; (60.8)	0.496	222	73.9 (67.6; 79.6)	<0.001
Household size <5 members, %	1,274	49.0 (46.3; 51.8)	1,052	46·3 (43.3; 49.4)	0.194	222	62.6 (55·; 68.9)	0.000
Paternal education: university, %	1,274	6.7 (5.4; 8.4)	1,052	6.7 (5.3; 8.4)	0.619	167	8.0 (3.0; 16.6)	0.020
Maternal education: no education, %	1,219	4.7 (3.5; 5.9)	1,052	3.7 (2.6; 4.9)	0.239	167	10.8 (6.1; 15.6)	<0.001

[1]Cut-off for having suspected CMD was a score of ≥6 points out of 20
[2]All prevalence and population proportions were estimated using weighted data
[3]Differences between proportions were tested by a two-sample test of proportions
[a] Prevalence comparison between completed dataset of the variables and final dataset (1,052)
[b] Prevalence comparison between excluded and included data
