# Supplementary material for: Determinants of Common Mental Disorders (CMD) among adolescent girls aged 15-19 years in Indonesia: Analysis of the 2018 National Basic Health Survey Data
Source: PLOS Glob Public Health. 2022 Mar 15;2(3):e0000232. doi: 10.1371/journal.pgph.0000232 (PMC10021533; doi:10.1371/journal.pgph.0000232)
Supplement: S3 Table — (PDF) [file pgph.0000232.s005.pdf]

S3 Table. Subdivision of CMD symptoms among girls aged 15-19 years old from Riskesdas Data 2018

Symptoms	Overall n (%)
**Depressive/anxious *(Yes)***	
01. Feel nervous, tense or worried	189 (17·8)
02. Easily frightened	161 (15·1)
03. Feel unhappy	83 (7·8)
04. Cry more than usual	86 (8·4)
**Somatic symptoms *(Yes)***	
05. Often have headaches	354 (32·6)
06. Sleep badly	246 (24·4)
07. Uncomfortable feeling in the stomach	161 (15·1)
08. Poor digestion	109 11·2)
09. Poor appetite	217 (20·8)
10. Hands shake	111 (10·8)
**Reduced vital energy *(Yes)***	
11. Easily tired	192 (19·3)
12. Difficult to make decisions	162 (15·4)
13. Difficult to enjoy your daily activities	66 (6·3)
14. Daily work suffering	41 (4·1)
15. Feel tired all the time	92 (9·1)
16. Trouble thinking clearly	106 (10·2)
**Depressive thoughts *(Yes)***	
17. Unable to play a useful part	36 (3·6)
18. Lost interest in things	65 (6·1)
19. Thoughts of ending your life	10 (1·0)
20. Feel that you are worthless person	52 (5·2)
