# Supplementary material for: Determinants of Common Mental Disorders (CMD) among adolescent girls aged 15-19 years in Indonesia: Analysis of the 2018 National Basic Health Survey Data
Source: PLOS Glob Public Health. 2022 Mar 15;2(3):e0000232. doi: 10.1371/journal.pgph.0000232 (PMC10021533; doi:10.1371/journal.pgph.0000232)
Supplement: S4 Table — (PDF) [file pgph.0000232.s006.pdf]

S4 Table. Eigen values of principal component in CMD

Items	Eigenvalue	Difference	Proportion	Cumulative
Component 1	2·2301	0·8407	0·1239	0·1239
Component 2	1·3894	0·1770	0·0772	0·2011
Component 3	1·2124	0·0392	0·0674	0·2684
Component 4	1·1732	0·0510	0·0652	0·333
Component 5	1·1222	0·0706	0·0623	0·3960
Component 6	1·0516	0·0582	0·0584	0·4544
Component 7	0·9934	0·0070	0·0552	0·5096
Component 8	0·9864	0·0109	0·0548	0·5644
Component 9	0·9756	0·0342	0·0542	0·6186
Component 10	0·9413	0·0121	0·0523	0·6709
Component 11	0·9292	0·0345	0·0516	0·7225
Component 12	0·8948	0·0363	0·0497	0·7722
Component 13	0·8585	0·0398	0·0477	0·8199
Component 14	0·8187	0·0299	0·0455	0·8654
Component 15	0·7888	0·0984	0·0438	0·9092
Component 16	0·6905	0·0814	0·0384	0·9476
Component 17	0·6092	0·2743	0·0338	0·9814
Component 18	0·3349	.	0·0186	1·0000
