# Supplementary material for: Determinants of Common Mental Disorders (CMD) among adolescent girls aged 15-19 years in Indonesia: Analysis of the 2018 National Basic Health Survey Data
Source: PLOS Glob Public Health. 2022 Mar 15;2(3):e0000232. doi: 10.1371/journal.pgph.0000232 (PMC10021533; doi:10.1371/journal.pgph.0000232)
Supplement: S5 Table — (PDF) [file pgph.0000232.s007.pdf]

S5 Table. Model fit of principal component regression

Component	Criterion of fit				
	F	RMSE	AIC	BIC	R^2
**Model 1**<br>(CMD: β0+β3pc3)	532·9*	2·727	5,108·7	5,118·7	0·337
**Model 2**<br>(CMD: β0+β3pc3+β2pc2)	267·8*	2·742	5,108·7	5,123·6	0·335
**Model 3**<br>(CMD: β0+β3pc3+β2pc1)	267·3*	2·746	5,109·4	5,124·3	0·335
**Model 4**<br>(CMD: β0+β3pc3+β2pc+β1pc1)	179·1*	2·741	5,109·3	5,129·3	0·336

*significant association (p<0·05)
